# YOLO_Bolt: a lightweight network model for bolt detection

**DOI:** 10.1038/s41598-023-50527-0

**Published:** 2024-01-05

**Authors:** Zhenyu Liu, Haoyuan Lv

**Affiliations:** Shenyang University of Technology, Shenyang, 110870 Liaoning China

**Keywords:** Mathematics and computing, Information technology

## Abstract

Accurate, fast, and intelligent workpiece identification is of great significance to industrial production. To cope with the limited hardware resources of factory equipment, we have made lightweight improvements based on You Only Look Once v5 (YOLOv5) and proposed a lightweight YOLO named YOLO_Bolt. First, ghost bottleneck lightweight deep convolution is added to the backbone module and neck module of the YOLOv5 detection algorithm to reduce the model volume. Second, the asymptotic feature pyramid network is added to enhance the feature utilization ability, suppress interference information, and improve detection accuracy. Finally, the relationship between the loss function and the decoupling head structure was focused on, and the number of decoupling head layers was redesigned according to different tasks to further improve the detection accuracy of the workpiece detection model. We conducted experimental verification on the MSCOCO 2017 dataset and the homemade bolt dataset. The experimental results show that compared with YOLOv5s, the number of model parameters is only 6.8 M, which is half that of the original model. On the MSCOCO 2017 dataset, the mAP increased by 2.4%. FPS increased by 104 frames/s. On the homemade dataset, the mAP 0.5 increased by 4.2%, and our proposed method is 1.2% higher than the latest YOLOv8s. The improved network can provide effective auxiliary technical support for workpiece detection.

## Introduction

Over the last 20 years, a number of complex industrial inspection problems have arisen due to the widespread use of certain workpieces, such as gears, bolts, bearings, etc., which require quality assurance. The conventional approach to workpiece inspection involves the use of manual inspectors at the final stage of the production line to perform random sampling for inspection purposes. However, the process of manual visual inspection is often tedious, time-consuming and prone to anomalies, resulting in an increase in defects due to workpiece mismatch. To mitigate this problem, an increased amount of duplicate work is generated, resulting in additional costs and longer detection times. It can therefore be argued that the implementation of automated part inspection systems has the potential to deliver significant economic benefits. The use of cameras and computers in automated visual inspection is designed to replace human vision in order to carry out evaluation and inspection tasks. Workpieces are evaluated and categorised, while defective ones are identified and rejected, based on many characteristics such as colour, scale and distance^1,2^.

The main objective of this study is to accurately and efficiently discriminate between different workpieces using machine vision technology. The YOLOv5 method is widely used in the YOLO family of algorithms due to its ability to achieve a balanced trade-off between detection speed and detection accuracy.

The research into target detection using YOLO has focused on two main areas: improving the model framework and processing the dataset. On the one hand, the YOLO series, starting with YOLOv3, features a feature pyramid^3,4^ that facilitates the prediction of objects at three different scales. This innovation significantly improves the ability to detect tiny targets. References^5,6^ add a detection layer to the basic YOLOv3 detection network, greatly improving its ability to identify small objects. Reference^7^ adds a direct link between the CBL layers of the two remaining units. The ability of the detection network to extract features was improved. By streamlining the feature extraction network and introducing a multi-layer series detection technique, Reference^8^ improves the efficiency and accuracy of feature extraction, these studies are all based on models to improve this direction. On the other hand, some scholars believe that YOLO requires large image data as a research driving force, the rare number of images lead to insufficient model fitting and affect the detection accuracy, so some researches started to study the processing of datasets, some of which were identified by extracting 3D point cloud data. Reference^9^ uses the advanced dynamic graph convolution neural network (DGCNN) for the partition of the point cloud of the coal mining workplace in the harsh environment, obtains the information of the coal cutting roof line, after the construction of multi-level serial polarization, the model shows good performance. Reference^10^ proposes a diversified generating model, which can generate high-quality and diversified images of alien objects on many intruding transmission lines, enriching the training datasets.

Although the above studies have significantly contributed to the improvement of the YOLO model as well as the accuracy of model detection, at present most of the studies focus on improving the detection accuracy and workpiece separation and image classification, while there are few studies on improving the detection speed and inference speed, and most of the studies do not include detailed data on the algorithm execution time. To solve the existing problem, this paper proposes a lightweight YOLOv5 model called YOLO_Bolt. Compared with standard YOLOv5, the model has reduced the number of parameters and the model's running cost, and the running speed is significantly reduced. The main contributions of this article are as follows:

1. The utilization of the better-performing ghost bottleneck^11^ replaces the bottleneck and convolutional network in the original backbone network, based on online transformation, obtaining richer target multi-channel characteristics, reducing the parameters and computational complexity of the network model, and improving the detection accuracy and efficiency to some extent.
2. Incorporate an asymptotic feature pyramid network (AFPN)^12^ to facilitate the direct exchange of non-adjacent levels, thereby mitigating the semantic information gap that exists between feature maps at different levels. This integration aims to improve the recognition accuracy of the model.
3. To improve the computational efficiency of the model's inference process, a novel lightweight asymmetric multilevel compression detector (LADH)^13^ has been proposed.

The rest of the paper is organised as follows: “Methodologies” describes in detail what we have done, including the techniques used and improved methods. The “Experimental studies” section introduces the experimental environment and details of the experimental deployment, as well as the evaluation indicators for analysing the experimental results. The “Results” section presents the experimental results. In the “Discussion” section, we discuss the experimental results. Finally, the conclusion and future work are presented in the “Conclusion” section.

## Methodologies

### Methods overview

**Figure 1:**
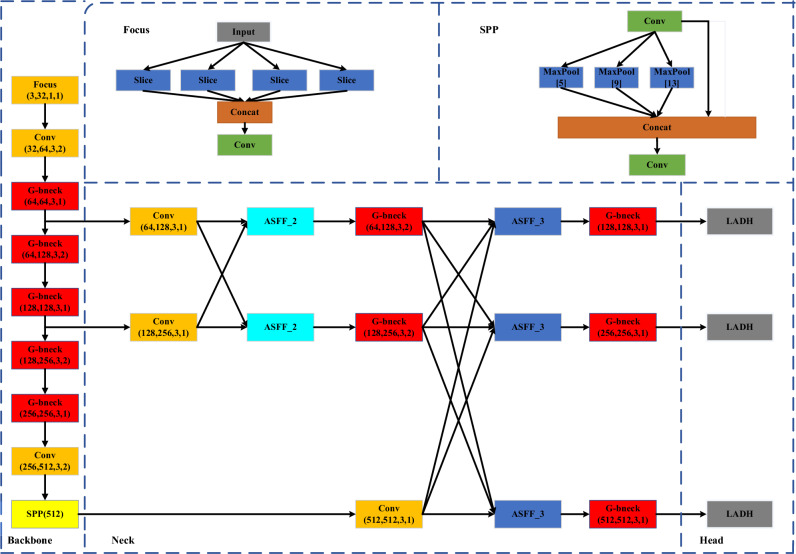
The structure of the proposed network.

The overall structure of the workpiece detection model proposed in this paper is shown in Fig. 1. This model is specifically designed for manufacturing environments and can be effectively implemented on mobile devices. The underlying architecture of the model is based on YOLOv5s, with enhancements made to the backbone network by exploiting the ghost bottleneck.

which is suitable for factory environments and can be deployed on mobile terminals. The entire model is based on YOLOv5s, and the backbone network is improved using the ghost bottleneck. In addition, we adopt an asymmetric feature pyramid network, to prevent significant changes in semantic information across feature maps at each level. This can lighten the computational load on the model while increasing its detection accuracy.Finally, a lightweight asymmetric multistage compression decoupling head is adopted. The novel decoupling head changes the structure of the head based on the correlation between the decoupling head structure and the loss function calculation logic, which in turn reduces the model computational load and increases the inference speed.

### Improvement of backbone network

The initial feature extraction network for YOLOv5s consists of 53 layers, which involves a significant number of parameters and computational operations. Furthermore, the performance of CSPDarknet53^14^ is constrained by the limitations of the conventional convolutional sampling technique, making it unable to effectively identify targets of varying sizes. To address the above issues, we choose to replace bottleneckCSP and Conv with ghost bottleneck.

**Figure 2:**
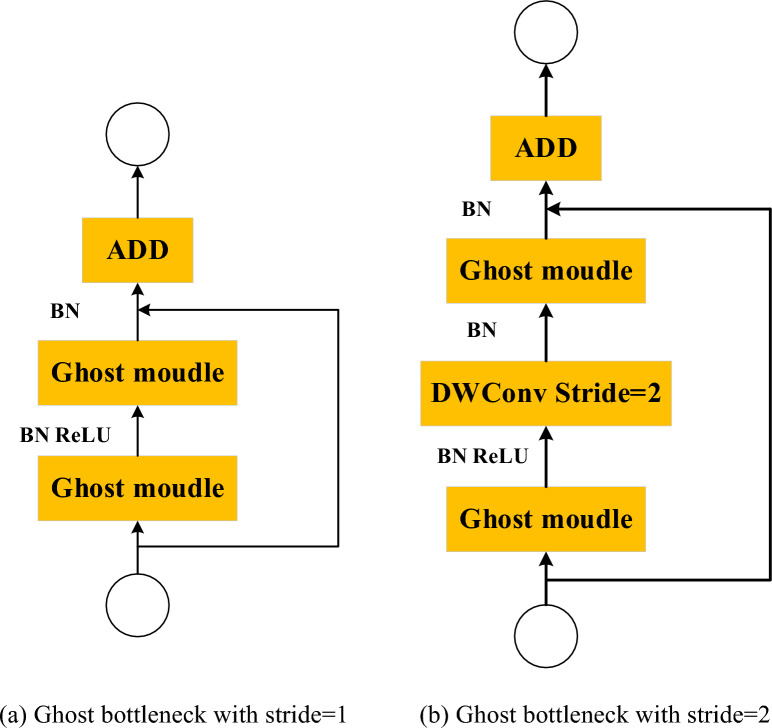
Ghost bottleneck.

The design of a ghost bottleneck is based on the features of the ghost module, as seen in Fig. 2. The ghost bottleneck (hereafter referred to as G-bneck) is influenced by the residual design found in the ResNet architecture. It includes numerous convolutional layers and a shortcut with two stacked modules to perform the G-bneck. However, the two ghost modules serve different purposes: the first module allows the number of data channels to be increased, while the second module allows the number of data channels to be reduced. In the case of G-bnecks with a stride of 1, the inclusion of batch normalization layers and ReLU activation functions follows the two ghost modules. The difference between G-bnecks with step 2 lies in the inclusion of a depthwise separable convolution with step 2, which is placed between the two ghost modules in order to accomplish the downsampling process.

In the backbone module, the bottleneckCSP and convolutional modules are replaced with ghost bottleneck modules, as illustrated in Fig. 1 (backbone). The bottleneck in the third layer is replaced with a Stride = 1 ghost bottleneck, keeping the output size of the model unchanged. The Conv layers in the fourth and sixth layers are replaced with Stride = 2 ghost bottlenecks for subsequent downsampling, and reduce dimensionality, decrease the number of parameters the network needs to learn, expand the receptive field, and prevent overfitting. The BottleneckCSP modules in the fifth and seventh layers are replaced with three ghost Bottleneck modules, all with Stride = 1.

### Improvement of the neck network

**Figure 3:**
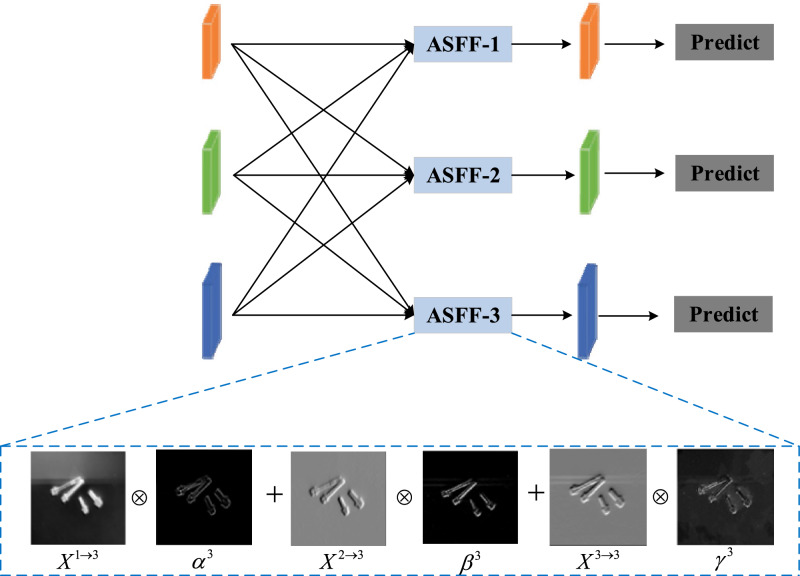
Illustration of the adaptive spatial feature fusion mechanism.

A common strategy for feature extraction at multiple scales is to use the well-established top-down and bottom-up feature pyramid networks. However, these methodologies suffer from the drawback of feature loss or degradation, which hampers the effectiveness of integrating non-adjacent levels. Therefore, we introduce an asymptotic feature pyramid network (AFPN) to support direct interaction at non-adjacent levels. Figure 3 illustrates the comprehensive structure of the asymptotic feature pyramid network (AFPN)^15^. Similar to most object identification techniques that rely on feature pyramid networks, the extraction of distinct layers of features from the backbone occurs prior to feature fusion. For the experiments conducted on the YOLOv5 framework, a convolution operation with a stride of 2 is applied on ${\text{P}}_{5}$, followed by another convolution operation with a stride of 1 to yield ${\text{P}}_{6}$. The ultimate collection of multiscale features consists of $\{{\text{P}}_{3}\text{,}{\text{P}}_{4}\text{,}{\text{P}}_{5}\text{,}{\text{P}}_{6}\}$, with corresponding feature strides of $\left\{6\text{4,}\text{ 12}\text{8,} \, \text{256,} \, {512}\right\}$ pixels.

The synthesis of the character map is based on the use of the attention mechanism concept, which assigns different weights to three different levels of character maps within a spatial context.

The AFPN is activated by the fusion of two adjacent low-level features, with higher-level features gradually integrated into the fusion process. By employing this approach, it is possible to mitigate the substantial semantic of disparity that exists between levels that are not directly contiguous. The synthesis of the character map is based on the use of the attention mechanism concept, which assigns different weights to three different levels of character maps within a spatial context.

#### Asymptotic architecture

During the feature extraction phase from the backbone network, the progressive feature pyramid incorporates features from several levels, including the bottom, middle, and top levels. It is worth noting that the top-level features are the most abstract among them. The disparity in semantics between layers that are not contiguous is more pronounced compared to the disparity between layers that are neighboring, particularly when considering the disparity between features at the lowest and highest levels. Consequently, there is a diminished fusion effect observed when integrating data obtained from layers that are not contiguous. Hence, it is not justifiable to employ $\{{C}_{3},{C}_{4},{C}_{5}\}$ for feature fusion. Therefore, in this study, a progressive feature pyramid approach is used to gradually integrate features. This methodology guarantees that the semantic information of features originating from various levels is brought together throughout the gradual fusion procedure, thus mitigating the problem of substantial discrepancies in semantic information across feature maps.

To align dimensions and prepare for feature fusion, we employ ${1}\times {1}$ convolutions and bilinear interpolation for upsampling of the feature maps. Conversely, for downsampling at the desired rate, we utilize different convolutional kernels and strides. Specifically, we apply a ${2}\times {2}$ convolution with a stride of 2 for two rounds of downsampling, a ${4}\times {4}$ convolution with a stride of 4 for four rounds of downsampling, and an ${8}\times {8}$ convolution with a stride of 8 for 8 rounds of downsampling.

#### Adaptive spatial fusion

We introduce adaptive spatial feature fusion (ASFF)^16^ to assign different spatial weights to features at different levels in the multilevel fusion process. This improvement highlights the significance of critical-level feature maps and reduces the impact of contradictory information originating from distinct objects. As shown in Fig. 3, We integrate feature map from three different levels. Let ${x}_{ij}^{n\to j}$ denote the feature vector at position $(i,j)$ from level *n* to level *l*. The resultant feature vector, denoted as ${y}_{ij}^{i}$, is obtained through the adaptive spatial fusion of multilevel features and is defined by the linear combination of feature vectors ${x}_{ij}^{1\to j}$, ${x}_{ij}^{2\to j}$, and ${x}_{ij}^{3\to j}$ as follows:

1 $$\begin{array}{c}{y}_{ij}^{i}={\alpha }_{ij}^{l}\cdot {x}_{ij}^{1\to j}+{\beta }_{ij}^{l}\cdot {x}_{ij}^{2\to j}+{\gamma }_{ij}^{l}\cdot {x}_{ij}^{3\to j}\end{array}$$

where ${\alpha }_{ij}^{l}$, ${\beta }_{ij}^{l}$, and ${\gamma }_{ij}^{l}$ represent the spatial weights of the features of the three levels at stage *l*, subject to the constraints that ${\alpha }_{ij}^{l}+{{\beta }_{ij}^{l}+\gamma }_{ij}^{l}=1$. To address the variation in the quantity of fused features at different stages of the Adaptive Feature Pyramid Network (AFPN), we have incorporated a stage-specific allocation of adaptive spatial fusion modules.

### Improvement of the head network

**Figure 4:**
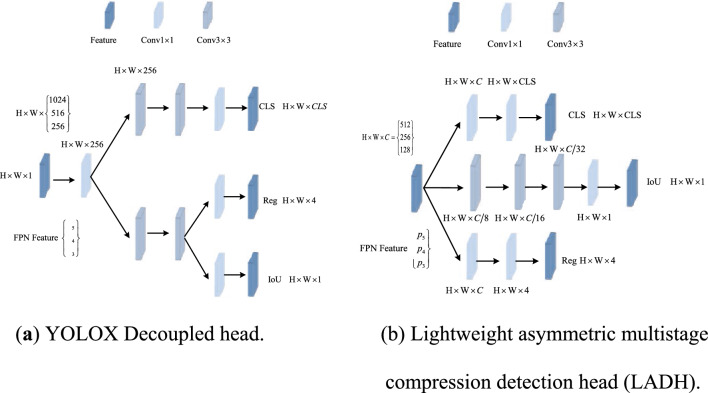
Comparison between Light Asymmetric Multilevel Channel Compression Decoupled Head and YOLOX's Decoupled Head.

The utilization of a decoupled head structure in YOLOX, as shown in Fig. 4a, is the initial occurrence of such a design inside the YOLO algorithm.The primary purpose of this structure is to address the conflict that arises between regression and classification tasks. The implementation of the decoupled head in YOLOX significantly enhances the detection accuracy of the model; however, it is not without notable concerns. Initially, it should be noted that the aforementioned architecture substantially augments the number of parameters and GFLOPs associated with the model, thereby leading to a reduction in inference speed. The proposed enhancements contradict the initial objective of developing a real-time object detection model.

A series of experiments were carry out to examine the concerns associated with the decoupled head in the YOLOX model. The results of our research show a relationship between the decoupled head and the corresponding loss functions. The loss function indicates that both positive and negative samples are involved in the calculation of objectness scores loss, but classification and bounding box loss focus solely on positive samples. Therefore, if the backpropagation loss value is calculated by the decoupled head model, it may cause the loss to be unevenly distributed if the network structure of classification, bounding box and object scale is the same. And this model may lead to the backpropagation of suboptimal error, thus preventing the achievement of gradient stability. Additionally, if we try to directly reduce the number of channels in the last layer to match the output channel, it may result in loss of features during forward transmission, thereby reducing network performance. The decoupled head should be adapted according to the computational difficulty of various jobs. In order to address these concerns, we introduce a unique decoupled head referred to as the lightweight asymmetric multilevel compression decoupled head, as shown in Fig. 4b.

In the proposed lightweight asymmetric multilevel compression detector, the network is partitioned according to different task types, and the corresponding tasks are executed across three distinct network paths. In this study, we focus on enhancing the path assigned to the item evaluation task by increasing the receptive fields and the number of parameters through the implementation of three convolutional layers. At the same time, the features of each convolutional layer are compressed in the channel dimension. This approach not only successfully mitigates the challenges associated with training the object scoring task, but also improves the performance of the model.

Moreover, it significantly reduces the parameters and GFLOPs of the decoupled head network, leading to a substantial improvement in the model's inference speed. We use a convolutional layer to separate the classification and localization tasks. For matched positive samples, the losses associated with these two tasks are relatively small. Therefore, we only employ one layer of convolution for separation to avoid over-expansion.

## Experimental studies

To verify the effectiveness and rationality of YOLO_Bolt in bolt target detection, we conducted two sets of experiments utilizing both the MSCOCO 2017 dataset and a self-built dataset for object detection.

1. Ablation experiment

The proposed YOLO_Bolt undergoes multiple stages of improvement. To verify the impact of each improvement strategy on the detection performance of the model, we designed ablation experiments on the MSCOCO 2017 dataset in this study.

(2) Comparative experiment

To further validate the detection performance of YOLO_Bolt on the object dataset, we selected four advanced object detection models for comparison.

### Dataset

#### MSCOCO 2017 dataset

MSCOCO 2017 refers to the 2017 version of the Microsoft Common Objects in Context Dataset. MSCOCO is a widely used computer vision dataset employed for tasks such as object detection, image segmentation, and human pose estimation.

#### Self-built bolt dataset

**Figure 5:**
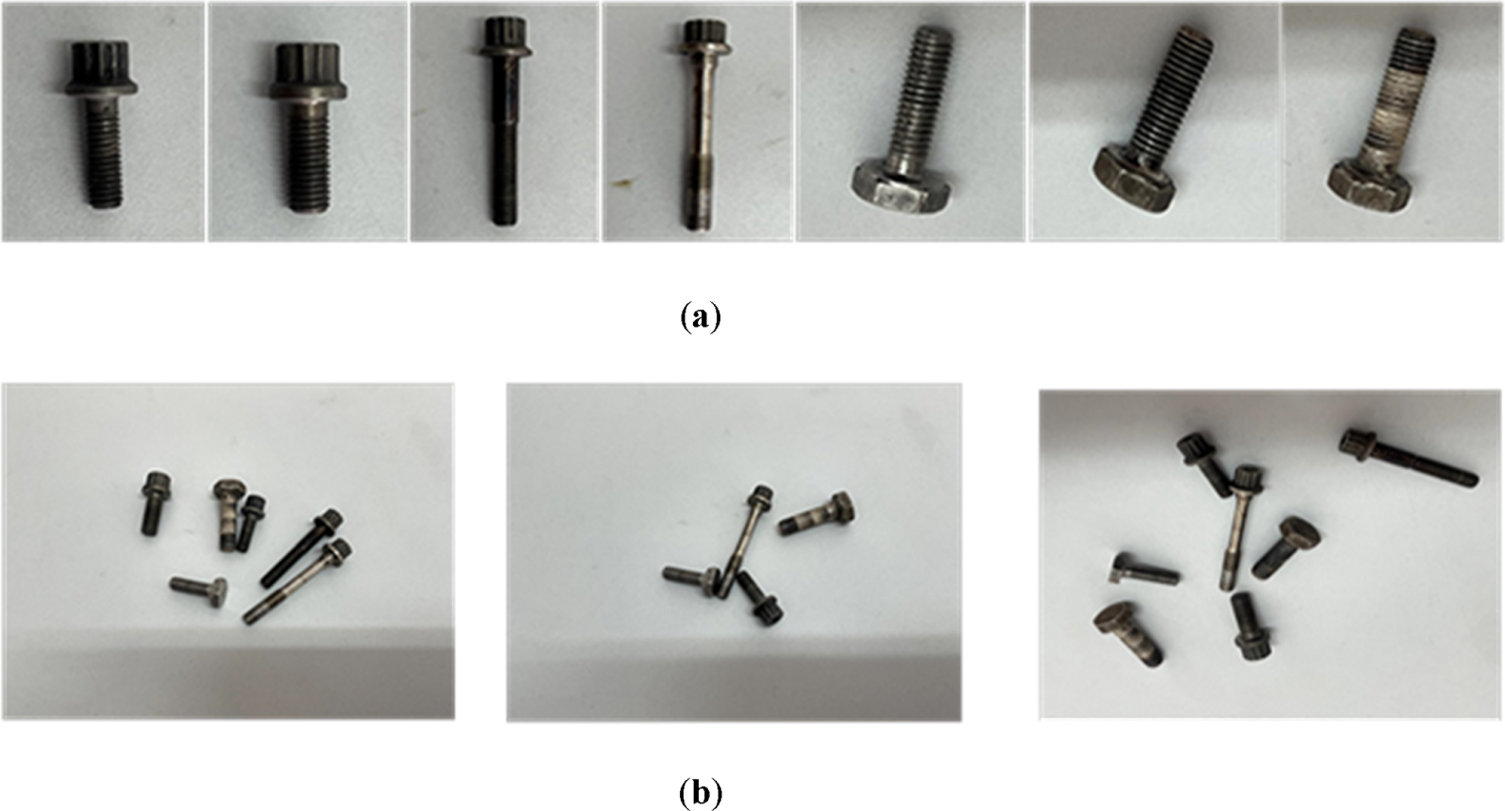
Examples of partial self-built dataset images. (**a**) From left to right are bolt_1-bolt_7; (**b**) detection sample example.

Since there is currently no publicly available large-scale dataset for industrial components, we created a dataset by photographing seven different bolt components. The camera of an iPhone 12 Pro (with the following specs: 12 MP, ultra wide-angle f/2.4 aperture and 120-degree viewing angle, wide-angle f/1.6 aperture, telephoto f/2.0 aperture) was used to shoot the image of the bolted with a 3042 × 4028 pixels resolution and the dataset, as partially shown in Fig. 5, consists of 2000 images for each type of component, totaling 14,000 pictures. These 10,000 images are allocated for the training set, 3,000 for the test set, and 1,000 for the validation set. Subsequently, we used the image annotation software labelimg to label the sample daya in the dataset, following a format consistent with the PASCAL VOC dataset. During the imaging process, the digital optometer GILTRON GT 1309 is utilised to regulate the light intensity within the range of 150–154 lx, and the camera is positioned at a fixed distance of 0.5 m in order to capture the desired image based on the detection environment at the factory.

### Experimental setup

#### Experimental platform

All experiments conducted in this study were carried out on a Windows 10 operating system utilizing an NVIDIA RTX 3090 24G GPU. The neural networks were constructed using PyTorch 1.7.1 as the fundamental framework. The training and testing datasets consisted of both the MSCOCO 2017 dataset and a self-built dataset for industrial work pieces.

#### Performance metrics

This study employs mean average precision (mAP) to assess the overall detection performance of the target detection across several types of targets, mAP is positively correlated with model detection performance. The speed of model detection is quantified by the rate at which images are frames per second (FPS).

2 $$\begin{array}{c}{\text{P}}{\text{r}}{\text{e}}{\text{c}}{\text{i}}{\text{s}}{\text{i}}{\text{o}}{\text{n}}\text{=}\frac{\text{TP}}{\text{TP+FP}}\end{array}$$

3 $$\begin{array}{c}{\text{R}}{\text{e}}{\text{c}}{\text{a}}{\text{l}}{\text{l}}\text{=}\frac{\text{TP}}{\text{TP+FN}}\end{array}$$

4 $$\begin{array}{c}AP={\int }_{0}^{1}{\text{Precision}}\left({\text{Recall}}\right)d\left({\text{Recall}}\right)\end{array}$$

5 $$\begin{array}{c}{\text{m}}{\text{A}}{\text{P}}\text{=}\frac{1}{{\text{N}}}\sum_{\text{i} = {1}}^{\text{N}}{{\text{AP}}}_{\text{i}}\end{array}$$

6 $$\begin{array}{c}{\text{F}}{\text{P}}{\text{S}}\text{=}\frac{1}{{\text{t}}}\end{array},$$

where true positive (TP) is the number of positive samples to be positive, and false positive (FP) is the number of samples predicted to be negative but actually positive. FPS means the pictures that can be processed per second, and *t* represents the time needed to process a picture.

#### Experimental details

During the training process, the detection model is trained using the stochastic gradient descent (SGD), with the batch size set to 32 and the training round epoch set to 100. The initial learning rate, weight attenuation and impulse factor are set to 0.001, 0.0005 and 0.9 correspondingly. The learning rate undergoes adjustment by a factor of 10 when the number of iterations exceeds 40,000, 60,000, and 80,000. Furthermore, the dataset for training the network is expanded by using several approaches, including image rotation, scaling, contrast correction, and saturation adjustment, to obtain additional examples of industrial components. This improvement in the network's generalisation capability led to better accuracy and stability in the detection of industrial workpieces.

The ablation experiments were mostly conducted on the MSCOCO 2017 dataset, as it is considered to have a higher reliability among public datasets. The YOLOv5s model was employed as the initial reference framework, followed by the incremental integration of the ghost bottleneck, AFPN, and LADH modules. We got the mean average precision (mAP) values for all targets in the dataset, along with the detection speed and model size, after each trial was completed. The influence of each improvement technique on the detection performance of the model was evaluated by observing the values of these metrics.

In comparative experiment, we conducted training and testing of YOLO_Bolt and many advanced detection algorithms using a dataset that we constructed ourselves. The operational conditions of each algorithm remain constant. Subsequently, the efficacy of the YOLO_Bolt model was also corroborated by the evaluation of metrics such as mean average precision (mAP), frames per second (FPS), and model size.

## Results

### Ablation experiment

The proposed YOLO_Bolt network introduces G-bneck into the backbone module and neck modules, and it incorporates a progressive feature pyramid structure to enhance the model's ability to integrate semantic information across multiple levels of feature maps. This compensates for the accuracy of loss incurred by lightweighting the backbone network. Additionally, we restructured the detection head, introducing a strategy to design decoupled heads for different task channels based on task type. To evaluate the efficacy of each enhancement, ablation experiments were performed on the MSCOCO 2017 dataset. To promote equity in the assessment process, we maintained consistent criteria for each variable.

Table 1 shows the comparative results of the ablation experiments when the input resolution is set to ${640}\times {640}$. The empirical results suggest that the module enhancement strategies presented in this study have a positive impact on the performance of the model. The baseline model used in this study is YOLOv5s, which incorporates the fundamental CSPDarknet53 backbone. The performance evaluation of this baseline model resulted in an mean average precision (mAP) of 44.8%.

**Table 1:** Ablation results.

Methods	Ghost	AFPN	LADH	mAP (%)	Para (M)	FPS (f/s)
Baseline	×	×	×	44.8	12.6	140
a	✓	×	×	42.3	10.2	164
b	✓	✓	×	46.7	6.4	203
c	✓	✓	✓	47.2	6.4	244

Method (a), which uses G-bneck improve the backbone network results in enhanced detection speed and improved detection accuracy in the model. The experimental results show that when comparing the modified YOLOv5s model with the original version, there was an improvement of 24 frames per second (FPS). Nevertheless, as a consequence of the drop in parameters inside the backbone network, there was a corresponding decline in the mean average precision (mAP) by a magnitude of 2.5%. This observation suggests that there is a lack of compatibility between the redesigned backbone network and the neck and head components of the original YOLOv5s architecture.

Method (b), which builds upon method (a), adds the asymptotic feature pyramid network. This further simplifies the network, reduces the number of model parameters while compensating for the detection accuracy of the model, enhences the importance of key-level feature maps, and suppresses the influence of interfering factors on the detection results, thereby enhancing the representational capability of object detection features. From the experimental results, it is evident that with the incorporation of the asymptotic feature pyramid network, the number of model parameters is reduced from 3.8 M to 6.4 M, FPS increased by 39 f/s, and mAP reached 46.7%, which fully compensated for the accuracy loss caused by method (a).

Method (c) optimises the decoupled head based on method (b). Modifying or replacing the loss function often affect the training process but does not directly impact the network inference time. This study investigates the relationship between the loss function and decoupled head. By altering the depth and width of the network based on the characteristics of each task and establishing different computational channels, the localization and classification capabilities of the detection model are further enhanced. The experimental results indicate that although the improvement in the decoupled head structure has a relatively minor impact on the mAP and the number of model parameters, it leads to a significant increase in the model's inference speed, achieving a boost of 31 f/s.

While the YOLOv5s exhibit the capability to detect distant and diverse bolt targets, it is important to acknowledge the possibility of encountering false detections. By replacing G-bneck and the asymptotic feature pyramid network, the occurrence of false detections is eliminated, resulting in a noticeable enhancement in the detection performance of the model.

As a result, all targets were successfully and precisely detected, our proposed methodology demonstrates superior detection efficacy in compared to YOLOv5.

### Comparison experiment

To further validate the overall detection performance of the improved YOLOv5s in the workpiece detection task, we compared the proposed YOLO_Bolt with four SOTA object detection algorithms. The compared algorithms are the original YOLOv5s, the region-based Faster R-CNN^17^, the regression-based SSD, and the latest YOLOv8 algorithm. To effectively compare the performance of the YOLO_Bolt algorithm, the training environment and dataset for all five algorithms were kept identical.

Based on the experimental results, we compared the performance of five models on our self-built dataset. The comparative results are shown in Table 2, which indicate that the proposed model achieves the highest values in terms of precision (Pr), recall (Re), average precision (AP), and mean average precision (mAP). It surpasses YOLOv5s by a margin of 4.2%. Given the lightweight nature of the model, which is designed to run on mobile hardware devices, we did not aim for higher FPS in order to conserve computing resources on the mobile devices.

**Table 2:** Comparison of evaluation index among different models.

Model	Pr	Re	mAP@0.5:0.95	mAP@0.5	FPS (f/s)	Para (M)
SSD	80.6	22.3	53.2	88.4	56.2	91.1
Faster R-CNN	84.3	42.6	58.4	81.7	7.8	338
YOLOv5s	86.7	82.4	63.0	94.3	140	14.4
YOLOv8s	85.3	84.7	72.3	97.4	277	11.2
Ours	91.2	84.3	75.6	98.6	107	6.8

Compared to Faster R-CNN, the proposed model in this paper shows an 16.9% improvement in mAP 0.5. However, there is a significant gap in terms of FPS, indicating that the two-step object detector is not suitable for industrial workspace detection tasks. When compared to SSD, there is a 10.2% improvement in mAP 0.5. Although SSD is a one-step detector, due to its inherent limitations, its detection accuracy does not yield satisfactory results, and its corresponding speed does not meet the requirements of industry workpiece detection tasks.

**Figure 6:**
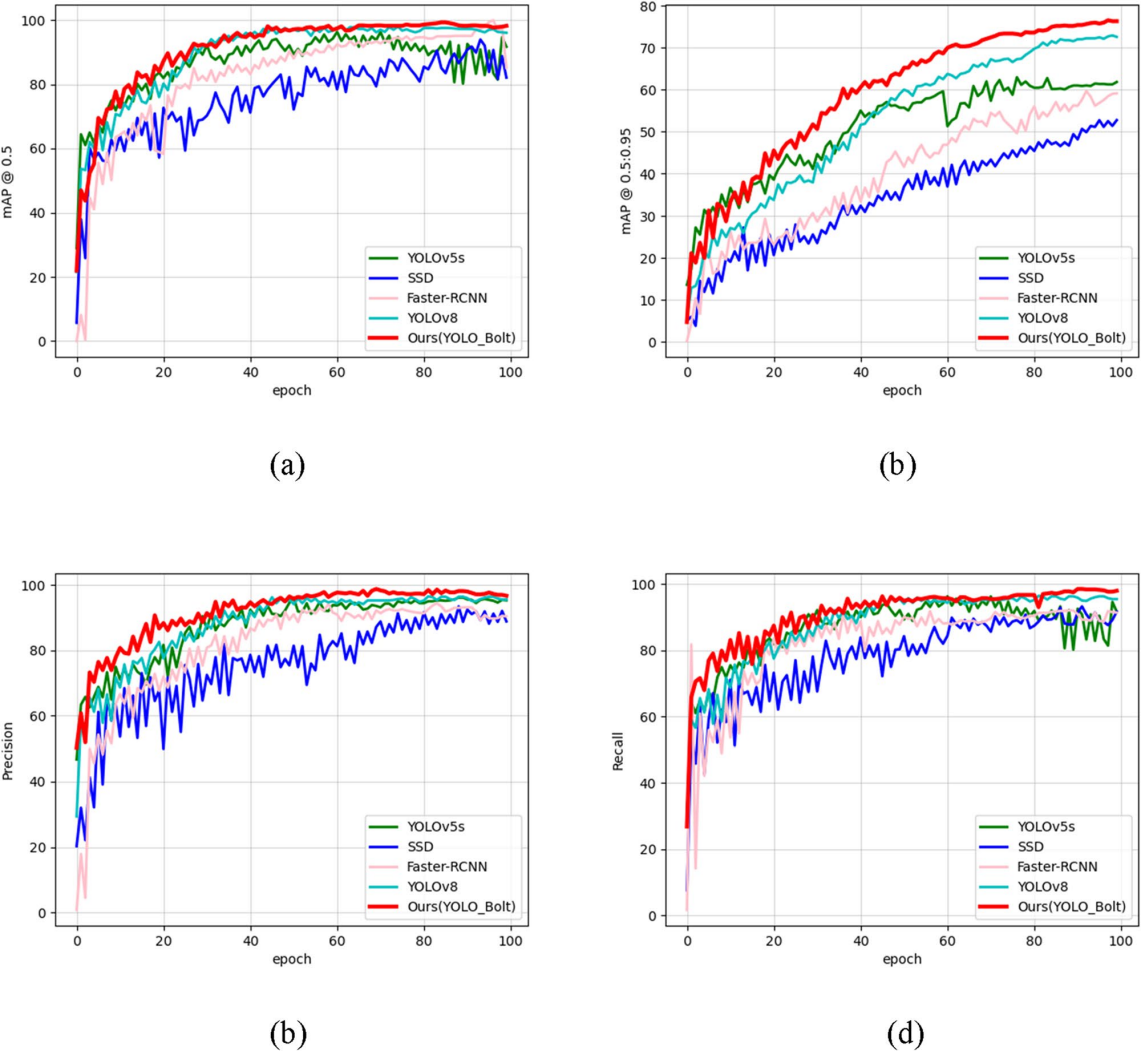
Comparison with other object detectors. (**a**) mAP 0.5 curve, (**b**) mAP 0.5:0.95 curve, (**c**) Precision curve, (**d**) Recall curve.

Figure 6c,d displays the accuracy and recall curves for several models. It is clear from looking at the comparison curve that the YOLO_Bolt model's curve gradually climbs without any apparent oscillations. In contrast, amplitude fluctuations of the curve are clearly visible in the rising stage of YOLOv5s and YOLOv8s, this phenomenon lasted until the middle and back of the model training. Figure 6a,b, and Table 2 all demonstrate that the YOLO_Bolt model has a greater mAP value than the rest of the models. mAP values of the enhanced model are 4.2% higher than those of the original model. All mAP 0.5 values of the YOLO_Bolt model are over 94%. The preceding diagram shows that YOLO_Bolt has received extensive training and that the fitting phenomenon has never happened (Supplementary Information).

The precision and recall curves of many models are depicted in Fig. 6c and d. The comparison curve reveals that the YOLO_Bolt model exhibits a gradual upward trajectory without significant fluctuation. Nevertheless, the YOLOv5s and YOLOv8s models are currently in the ascending phase of the curve, exhibiting noticeable fluctuations in amplitude throughout the whole duration of the curve. According to the findings presented in Fig. 6a and b, and Table 2, it is evident that the mean average precision (mAP) value of the YOLO_Bolt model surpasses that of the other models. The mAP 0.5 value for the YOLO_Bolt model consistently exceeds 94%. Furthermore, the improved model demonstrates a 4.2% increase in mAP compared to the pre-improved model. The figure presented above demonstrates that YOLO_Bolt has undergone thorough training and has not exhibited any instances of fitting phenomena.

**Figure 7:**
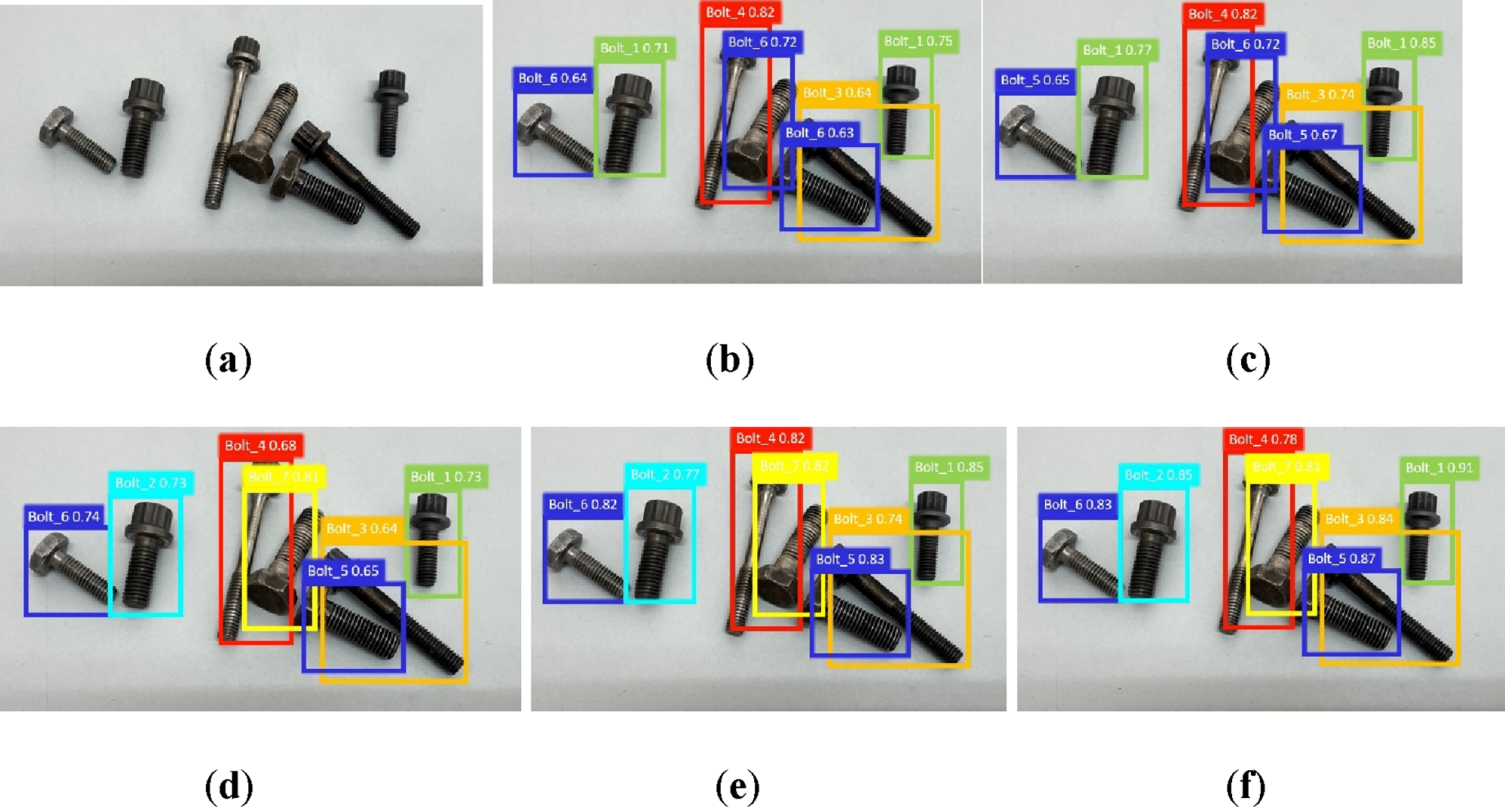
Comparison of detection of different models in example 2. (**a**) The original image; (**b**) SSD; (**c**) Faster R-CNN; (**d**) YOLOv5s; (**e**) YOLOv8s; (**f**) YOLO_Bolt.

**Figure 8:**
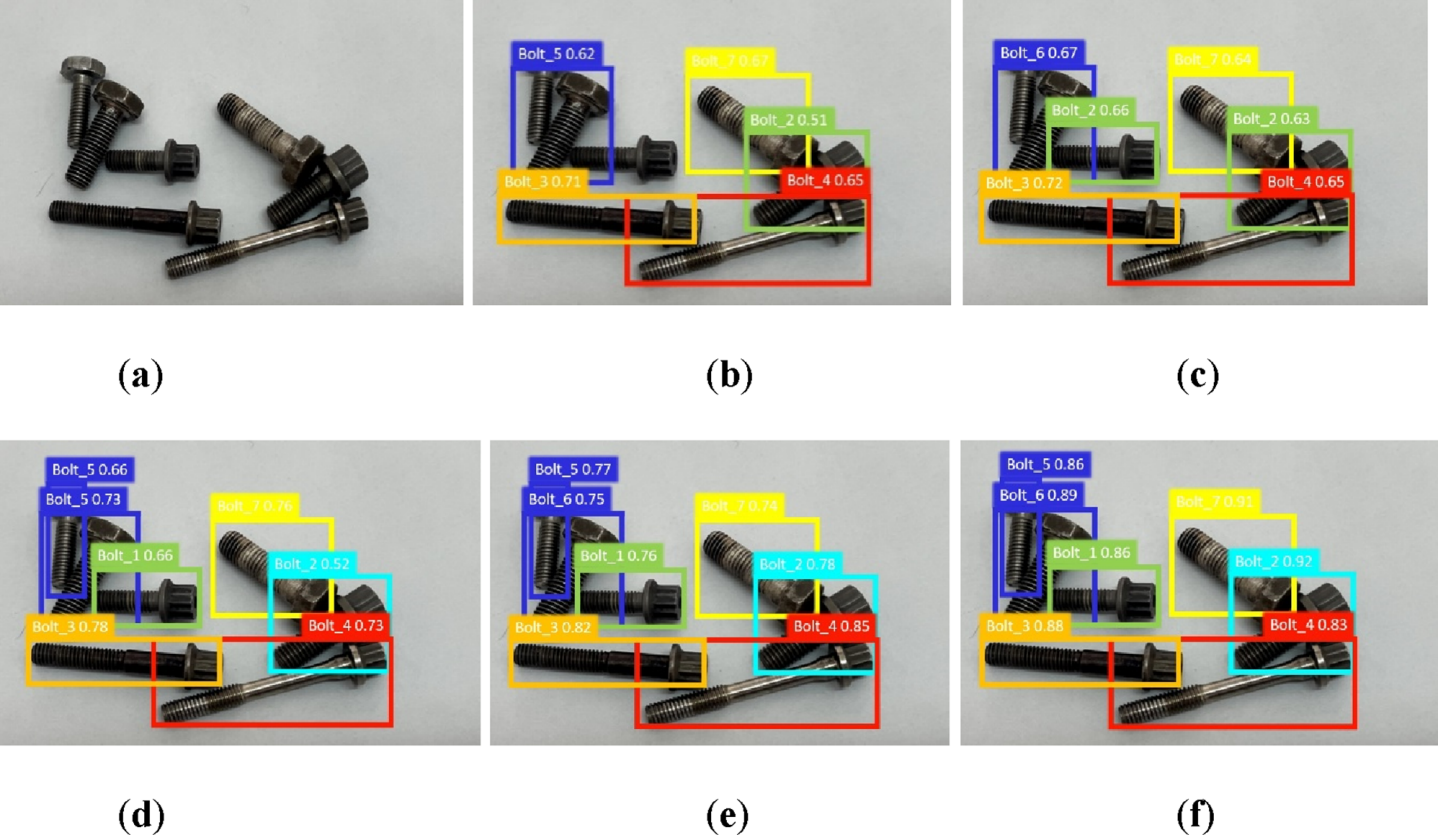
Comparison of detection of different models in example 3. (**a**) The original image; (**b**) SSD; (**c**) Faster R-CNN; (**d**) YOLOv5s; (**e**) YOLOv8s; (**f**) YOLO_Bolt.

In order to fully illustrate the applicability of our model to different examples, we give the target detection results of several typical examples in Figs. 7 and 8a–f. These figures show the detection results obtained from the original image, SSD, Faster R-CNN, YOLOv5s, YOLOv8s, and our approach, respectively.

From the detection result of the example, it can be seen that the detection result of SSD is worst, with a dense bolt undetected and two false detections. The detection results of YOLOv5s and YOLOv8s are similar, but their accuracy is not satisfactory. YOLO_Bolt can fully identify all targets with high accuracy. In simple scenes, Faster R-CNN has better detection on Bolt_4 and Bolt_5, second to our model. YOLO_Bolt is the best among several models, not only effectively identifying all kinds of targets, but also having high detection confidence.

Based on the current results, YOLOv8, as the latest algorithm in the YOLO series, achieves an mAP closest to ours without any specific improvements. However, the proposed method still has an advantage in that its detection speed is sufficient to meet the practical needs of industrial workpiece detection tasks. In terms of model size, our proposed model is reduced to half the size of the original YOLOv5s, thus realizing a lightweight network.

Therefore, based on the results of the above experiments, compared to other algorithms, our approach has improved detection accuracy and speed. The detection performance of our proposed method is the best, which provides valuable information for lightweight deployment in industrial workpiece detection tasks.

## Discussion

In industrial production environments, workpiece testing poses several challenges. Firstly, the process is characterized by high real-time demands, as testing must be carried out quickly and efficiently. Additionally, the volume of workpieces to be tested is often substantial, necessitating the ability to handle large amounts effectively. Moreover, workpiece testing typically involves a significant investment of time due to the complexity involved. Lastly, the inherent instability of workpieces can introduce the risk of disinformation or data loss, further complicating the testing process.

Traditional object detection algorithms require extensive preprocessing of images before detection, involving tasks such as correcting distorted images captured by cameras, followed by pixel traversal, threshold comparisons, and grayscale operation on the entire image, which take a long time.

In the ablation experiments, the mAP of the model increased by 2.4% on the MSCOCO 2017 dataset and by 9.3% on the self-built bolt datasets. This significant increase in FPS allows YOLO_Bolt to maintain high-precision detection of bolt targets while achieving a high detection speed. This will help to find the targets faster and more accurately and lay the foundation for the next step of robotic arm grasping and softening.

In the ablation experiment, the mAP of the model increased by 2.4% on the MSCOCO 2017 dataset, up 4.2% for the self-built workpiece dataset. The significant increase in FPS enables YOLO_Bolt to maintain high-precision detection of screw targets while achieving high detection speeds.This will help to find targets faster and more accurately, laying the foundation for the next step of mechanical arm grinding and softening.

In the comparative experiments of several algorithms, YOLO_Bolt exhibited superior performance in terms of detection accuracy metrics. It closely trailed the most recent one-step detector YOLOv8, Compared to Faster R-CNN, SSD, YOLOv5s, and YOLOv8s with notable enhancements in mAP 0.5 values of 16.9%, 10.2%, 4.3%, and 1.2% respectively. In general, the performance of YOLO_Bolt continues to be the most superior.

Considering that the workpiece defects of manufacturing have particularly high requirements for real-time performance, current production lines require the identification of major categories of objects within 3 s. These objects are then directed to corresponding areas for further sorting. If the detection cannot be completed within this 3 s, the object may bypass the classification gate, leading to missed detections. The proposed detection algorithm not only meets these requirements but also exceeds the accuracy demands, far outperforming traditional algorithms in detection speed. Currently, due to the globalization of industrial production, there is wide variation in the quality of raw materials and processing conditions, resulting in differences in the color and surface texture of objects in each batch. This model effectively identifies and categorizes the types of objects in each batch. This model effectively identifies and categorizes types of objects, overcoming factors such as varying conditions and materials. The model demonstrates strong generalization capabilities and does not require pa-ramete adjustments for different batches. In contrast, traditional object detection algorithms need to readjust the threshold parameters through the workpiece quality of each batch and f-actory environment. Adjusting the threshold is a complex process that greatly reduces the detection efficiency.

## Conclusion

This article constructs a workpiece detection dataset for Workpiece detection tasks in a factory environment. To adapt to the limited hardware resources of factory detection equipment, we lightweight the original YOLOv5s model. An improved target detection method based on YOLO have been proposed. In our study, the utilizition of a lightweight feature extraction network reduces the parameters and computation of the model, thus improving the detection performance. By introducing an Asymptotic Feature Pyramid structure, the model's ability to use semantic information of feature maps is improved. Finally, the relationship between the loss function and the decoupled head network was studied, and the network structure of the classification and positioning tasks was modified according to the workpiece detection task to further improve the detection accuracy of the bolt target. Through ablation experiments and comparison experiments, it is proved that YOLO_Bolt has better detection performance for bolts and can meet the detection requirements in factory situations.

However, the application scope of the improved model are still limited. First of all, for the dataset, the current workpiece type are limited, there are only seven kinds of bolt workpiece in the dataset. There are also workpiece image data such as gaskets, nuts, silencers, ets. that have not been collected. In order to improve the accuracy of the algorithm and application scope, while the system is applied for a long time in the future, on the one hand, the workpiece sample database will be established, on the other hand, a defect sample database corresponding to the workpiece will be eatablished, and the improved dataset is added to the later training model to train a more complete detection model. For the model, the current model is not ideal for detecting severely overlapping workpieces. It cannot detect the effective features of the obscured workpieces. It can only rely on the robotic arm to sort out the unblocked workpieces first, and after the bottom workpieces are exposed, they are sent to the next detection point.m which will prolong the length of the assembly line and cause unnecessary costs. In addition, we will use the improved strategy in this paper to improve YOLOv7 and YOLOv8 and verify the improvement effect on the MSCOCO 2017 dataset and our self-built dataset. We will further optimize the detection model proposed in this paper and deploy it on embedded platforms to verify the suitability of the testing model in devices with limited hardware resources.

### Supplementary Information

Supplementary Information.

## Data Availability

Data or code presented in this study are available request from the corresponding author.
